# Effect of community members' willingness to disclose a mental disorder on their psychiatric symptom scores: analysis of data from two randomised controlled trials of mental health first aid training

**DOI:** 10.1017/S2045796019000404

**Published:** 2019-08-09

**Authors:** A. F. Jorm, A. J. Mackinnon, L. M. Hart, N. J. Reavley, A. J. Morgan

**Affiliations:** 1 Centre for Mental Health, Melbourne School of Population and Global Health, University of Melbourne, Melbourne, Australia; 2 Black Dog Institute, University of New South Wales, Sydney, Australia

**Keywords:** Adolescents, attitudes, common mental disorders, mental health, stigma

## Abstract

**Aims:**

The prevalence of common mental disorders has not declined in high-income countries despite substantial increases in service provision. A possible reason for this lack of improvement is that greater willingness to disclose mental disorders might have led to increased reporting of psychiatric symptoms, thus masking reductions in prevalence. This masking hypothesis was tested using data from two trials of interventions that increased willingness to disclose and that also measured symptoms. Both interventions involved Mental Health First Aid (MHFA) training, which is known to reduce stigma, including unwillingness to disclose a mental health problem.

**Methods:**

A cross-lagged panel analysis was carried out on data from two large Australian randomised controlled trials of MHFA training. The first trial involved 1643 high school students in Year 10 (mean age 15.87 years), who were randomised to receive either teen MHFA training or physical first aid training as the control. The second trial involved 608 Australia public servants who were randomised to receive either eLearning MHFA, blended eLearning MHFA or eLearning physical first aid as the control. In both trials, willingness to disclose a mental disorder as described in vignettes and psychiatric symptoms (K6 scale) were measured pre-training, post-training and at 12-month follow-up.

**Results:**

Both trials found that MHFA training increased willingness to disclose. However, a cross-lagged panel analysis showed no effect of this change on psychiatric symptom scores.

**Conclusions:**

Greater willingness to disclose did not affect psychiatric symptom scores. Because the trials increased willingness to disclose through a randomly assigned intervention, they provide a strong causal test of the masking hypothesis. It is therefore unlikely that changes in willingness to disclose are masking reductions in prevalence in the population.

## Introduction

Data from a number of high-income countries show that, despite substantial increases in the provision of treatment over time, there has not been any reduction in the prevalence of common mental disorders or psychiatric symptoms (Ormel *et al*., 2004; Jorm *et al*., 2017; Mulder *et al*., 2017; Bastiampillai *et al*., 2019). Prevalence rates have similarly been stable for children and adolescents, apart from an increase in depression in adolescent girls and possibly in boys (Bor *et al*., 2014). Jorm *et al*. (2017) argued that reducing prevalence may require a greater emphasis on the quality of services and on prevention. However, they also considered the hypothesis that a reduction in prevalence produced by services may have been masked by increased reporting of symptoms due to greater public awareness of common mental disorders or reduction in stigma leading to a greater willingness to disclose. At the time of their review, there were no relevant data to test this masking hypothesis.

Here we report data from two randomised controlled trials that were used to test the masking hypothesis. Both trials involve Mental Health First Aid (MHFA) courses. These courses train members of the public in how to offer help to a person developing a mental health problem, experiencing a worsening of an existing mental health problem or in a mental health crisis (Jorm *et al*., 2019). A systematic review and meta-analysis of MHFA trials showed that these courses increase mental health knowledge and reduce stigma (Morgan *et al*., 2018). Data from the first trial used here were on teen MHFA training of students in Australian high schools (Hart *et al*., 2018). In a cluster-randomised cross-over design, schools were randomised to receive either teen MHFA or physical first aid. The other dataset was a randomised controlled trial of MHFA training with staff in Australian workplaces, which compared eLearning and blended modes of MHFA (i.e., eLearning combined with a classroom session) with eLearning physical first aid (Reavley *et al*., 2018). Both trials asked questions about willingness to disclose mental health problems and also measured psychiatric symptoms both before and after training. We analysed these data using cross-lagged panel analysis to test the hypothesis that an increase in willingness to disclose leads to increased reporting of psychiatric symptoms.

## Methods

### Adolescent MHFA trial

This trial was registered with the Australian New Zealand Clinical Trials Registry (ANZCTRN12614000061639) and the details have been previously reported (Hart *et al*., 2018). In a cluster-randomised crossover design, four Australian public schools were matched in two pairs and then randomised to either receive teen MHFA or physical first aid for all Year 10 students (mean age 15.87 years). The following year, the new Year 10 cohort received the alternate intervention, giving a total of eight cohorts. Classes comprising 979 students were randomised to teen MHFA and classes with 948 students to physical first aid. Not all students participated in the trial, yielding responses from 1643 individuals. The effects of the training were assessed using online surveys which were administered pre-training, 1-week post-training and at 12-month follow-up. The trial was carried out from 2014 to 2017.

There were two outcome measures of relevance to the masking hypothesis. The first was a question asked in relation to vignettes of an adolescent with major depression and suicidal ideation (John) and one with social anxiety disorder (Jeanie). The vignettes (see Appendix) described signs and symptoms consistent with Diagnostic and Statistical Manual of Mental Disorders (DSM) diagnostic criteria, but did not involve any diagnostic labelling of the person's problem. The statement ‘If I had a problem like (John/Jeanie)'s I would not tell anyone’ was rated on a five-point Likert scale ranging from 1 = ‘strongly agree’ to 5 =  ‘strongly disagree’ (Griffiths *et al*., 2004, 2006; Yap *et al*., 2014). This question was asked at all three time points. The two vignette ratings at each time point were summed to give a measure of willingness to disclose. The second measure was the K6 psychological distress scale, which asks about symptoms in the previous 30 days (Kessler *et al*., 2002). This scale was administered pre-training and at follow-up, but not post-training.

### Adult MHFA trial

This trial was registered (ACTRN12614000623695) and the details have been previously reported (Reavley *et al*., 2018). Participants were 608 Australian public servants who were individually randomised to receive either eLearning MHFA (*n* = 199), blended eLearning MHFA (*n* = 199) or eLearning physical first aid as the control group (*n* = 210). For the purposes of the present analysis, both MHFA groups were combined and compared with the control group. Outcomes were assessed by online questionnaire pre-training, post-training and at 12-month follow-up.

Again, there were two outcome measures of relevance here. The first was the disclosure question, which was asked in relation to a vignette of an adult with major depression and suicidal thoughts (John) and one with post-traumatic stress disorder (Paula), with the responses summed. As for the adolescent study, the vignettes described signs and symptoms consistent with DSM diagnostic criteria, but did not give any diagnostic label (see Appendix). These disclosure questions were administered at all three time points. The K6 was also administered pre-training and at follow-up. The trial was carried out (up to 12-month follow-up) from 2014 to 2018.

### Data analysis

**Fig. 1.**
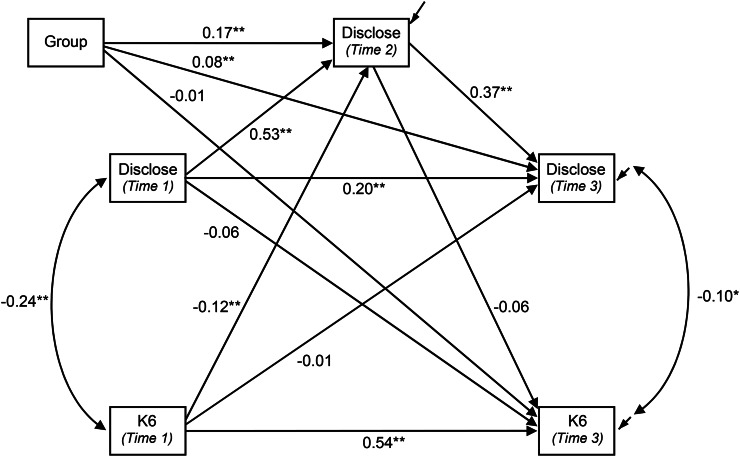
Cross-lagged model of willingness to disclose a mental disorder (Disclose) and K6 score in adolescents. (Paths from Group are standardised coefficients for dependent variable only: all others are fully standardised. *: *p* < 0.05; ** *p* < 0.01 established from bootstrap confidence intervals.)

**Fig. 2.**
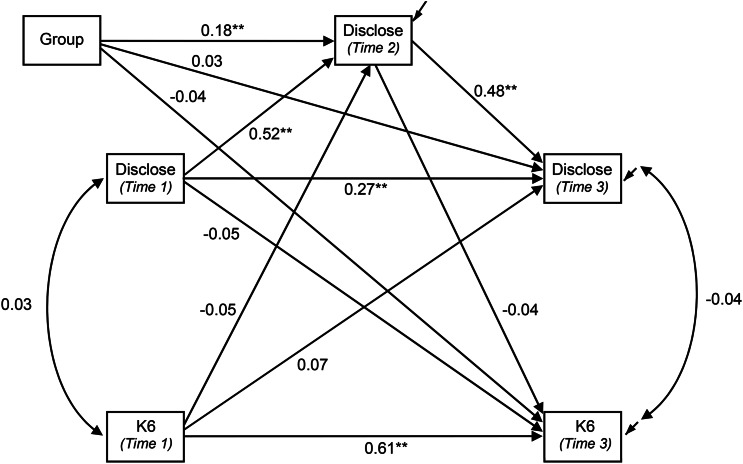
Cross-lagged model of willingness to disclose mental disorder (Disclose) and K6 score in adults. (Paths from Group are standardised coefficients for dependent variable only: all others are fully standardised. * *p* < 0.05; ** *p* < 0.01 established from bootstrap confidence intervals.)

The model fitted to both datasets was a cross-lagged design with additional paths to accommodate the additional assessment of willingness to disclose at an intermediate point between baseline and each trial's endpoint (see Figs 1, 2). The outcomes of both the K6 and willingness to disclose were expected to be substantially determined by the status of each variable earlier in the trial. Principal interest lay in the cross-lagged paths (i.e., the influence of earlier values of willingness to disclose on subsequent K6 scores and vice versa). Supplementary analyses showed the effect of eLearning and blended eLearning MHFA were highly comparable. Accordingly, the effects of the MHFA training intervention were modelled by the inclusion of a single binary indicator (active arm (either MHFA course) = 1; control = 0) with paths to each outcome. Due to randomised assignment to trial group, this variable was specified as being independent of baseline values of the K6 and of willingness to disclose. Model fit was assessed using the Tucker Lewis Index (TLI), Comparative Fit Index (CFI) and the Root Mean Square Error of Approximation (RMSEA). Values of the first two indices greater than 0.95 were taken as demonstrating good fit, as were values of RMSEA <0.06 (Hu and Bentler, 1999). The significance of individual paths and indirect and total effects was assessed using confidence intervals constructed from 10 000 bootstrapped samples. This approach was adopted due to non-normal distributions of the variables in the model and the need to assess the significance of indirect and total effects. Parameters whose 99% confidence intervals excluded zero were considered significant at the equivalent of *p* < 0.01, while those whose 95% confidence interval excluded zero were deemed significant at *p* < 0.05. Models were fitted using Mplus version 7.4 (Muthén and Muthén, 1998–2015). With the adolescent MHFA trial, clustering of participants within schools was not accommodated in the model, as this effect was not significant in primary analyses (Hart *et al*., 2018) and Mplus does not support bootstrapping of clustered samples.

## Results

### Adolescent MHFA trial

The model in Fig. 1 was fitted to 1643 participants in this trial. Descriptive statistics for the variables in the model are shown in Table 1. The model provided an excellent fit to the data (*χ*^2^ = 7.00, df = 2, *p* = 0.030; RMSEA = 0.039, *p* close fit = 0.658; CFI = 0.99, TLI = 0.97).

**Table 1.** Descriptive statistics for willingness to disclose and K6 for each occasion of measurement by trial and intervention status

	*Time 1* Mean (s.d.)	*Time 2* Mean (s.d.)	*Time 3* Mean (s.d.)
*Adolescent MHFA trial*			
Willingness to disclose			
Control	3.51 (0.96) *n* = 769	3.52 (0.98) *n* = 560	3.44 (0.95) *n* = 409
MHFA	3.61 (0.96) *n* = 792	3.89 (0.90) *n* = 524	3.73 (0.88) *n* = 446
K6 Total score			
Control	13.82 (5.47) *n* = 757	—	13.89 (5.28) *n* = 399
MHFA	13.27 (5.23) *n* = 778	—	13.36 (5.05) *n* = 433
*Adult MHFA trial*			
Willingness to disclose			
Control	4.17 (0.75) *n* = 210	4.06 (0.90) *n* = 77	4.18 (0.78) *n* = 83
MHFA	4.11 (0.79) *n* = 398	4.33 (0.60) *n* = 207	4.26 (0.72) *n* = 182
K6 Total score			
Control	14.02 (5.97) *n* = 210	—	14.20 (6.35) *n* = 79
MHFA	13.78 (5.79) *n* = 398	—	13.43 (6.10) *n* = 181

Figure 1 shows the expected pattern of substantial and significant effects that earlier values of each variable have on their subsequent status. The inclusion of a direct path from Time 1 to Time 3 values of willingness to disclose led to a significant improvement in fit (Δ*χ*^2^ = 26.84, df = 1, *p* < 0.001) and so was retained in the model.

Consistent with the mixed model analysis reported previously (Hart *et al*., 2018), the teen MHFA training led to a significant increase in willingness to disclose at both outcome occasions. The direct effect of training on K6 scores was negligible and non-significant, as was the indirect effect via willingness to disclose at Time 2 (standardised effect: −0.01; 95% CI: −0.02–0.01). Consequently, the overall effect of group on K6 was not significant (see Table 2).

**Table 2.** Total effects of baseline status on outcomes in the adolescent MHFA trial

Outcome	Predictor	Standardised effect	95% CI
Willingness to disclose (Time 3)			
	Willingness to disclose (Time 1)	0.40**	0.33–0.47
	K6 (Time 1)	−0.05	−0.12–0.02
	Group (MHFA *v*. control)	0.14**	0.08–0.20
K6 (Time 3)			
	Willingness to disclose (Time 1)	−0.09**	−0.15 to −0.03
	K6 (Time 1)	0.55**	0.49–0.60
	Group (MHFA *v*. control)	−0.02	−0.08–0.02

* *p* < 0.05; ** *p* < 0.01.

Table 2 shows that the total effect of baseline K6 score on willingness to disclose at Time 3 was small and non-significant. However, the direct effect of baseline K6 on willingness to disclose at Time 2 and the indirect effect alone via willingness at Time 2 (standardised effect: −0.04; 95% CI: 0.02–0.07) were both statistically significant.

### Adult MHFA trial

The model developed for the adolescent sample was fitted to data from 608 participants in the adult trial. Descriptive statistics for the variables in the model are shown in Table 1. The model yielded comparable results to the adolescent sample. Once again, model fit was excellent (*χ*^2^ = 1.09, df = 2, *p* = 0.579; RMSEA = 0.000, *p* close fit = 0.868; CFI = 1.00, TLI = 1.00). As expected, each variable was substantially influenced by its previous value. The direct path from willingness to disclose at baseline to Time 3 was also significant (Δ*χ*^2^ = 20.61, df = 1, *p* < 0.001).

The effect of intervention group on willingness to disclose was significant at Time 2. The direct effect of the intervention on willingness to disclose at Time 3 was not significant (see Fig. 2) but the total effect, mainly via willingness to disclose at Time 2, was significant (see Table 3). Neither the direct effect of the intervention on the K6 nor the total effect was significant. All cross-lagged effects of baseline K6 and willingness to disclose on each other were small and non-significant.

**Table 3.** Total effects of baseline status on outcomes in adult the MHFA trial

Outcome	Predictor	Standardised effect	95% CI
Willingness to disclose (Time 3)			
	Willingness to disclose (Time 1)	0.52**	0.40–0.62
	K6 (Time 1)	0.05	−0.06–0.16
	Group (MHFA *v*. control)	0.11*	0.01–0.22
K6 (Time 3)			
	Willingness to disclose (Time 1)	−0.06	−0.15–0.03
	K6 (Time 1)	0.61**	0.49–0.60
	Group (MHFA *v*. control)	−0.05	−0.14–0.04

* *p* < 0.05; ** *p* < 0.01.

## Discussion

Both trials found that MHFA training increased willingness to disclose a mental disorder. However, the cross-lagged analysis found no effect of this increase on K6 score in either trial. While willingness to disclose was measured pre-training, post-training and at 12-month follow-up, K6 score was only measured pre-training and at 12-month follow-up. The reason for not also administering the K6 post-training is that the questions refer to the past 4 weeks, which overlaps with the period of the training. It was not thought to be plausible that any change could occur over this time frame. It could be argued that 12 months is too long to wait to assess the impact of changes in willingness to disclose and that ideally there should have been an intermediate measurement point. While an intermediate measure would have been desirable, both trials did find an impact of MHFA training on willingness to disclose at the 12-month follow-up, providing an adequate test of the masking hypothesis.

These analyses have a number of strengths. Because the trials increased willingness to disclose through a randomly assigned intervention, they provide a strong causal test of the hypothesis that willingness to disclose increases psychiatric symptom scores. The finding of no link between willingness to disclose and symptom scores is also strengthened by the replication across two different age groups. The sample sizes were also relatively large (1643 and 608 participants), giving good statistical power to detect small effects.

However, there are also limitations. The measure of willingness to disclose was brief and consequently had modest reliability. The vignettes also covered a limited range of mental disorders (major depression with suicidal thoughts and social anxiety disorder for adolescents, and major depression with suicidal thoughts and post-traumatic stress disorder for adults). Although the K6 is a well-validated questionnaire, it covers a limited range of symptoms. There could be different results for more stigmatised symptoms (e.g. suicidality, thought disorders). On the other hand, the K6 is an appropriate questionnaire for evaluating the masking hypothesis, because it covers symptoms of the most prevalent mental disorders and there is excellent Australian population data showing no historical change on the K10 (the parent questionnaire of the K6) despite major increases in use of services (Jorm, 2018, 2019).

Another limitation is that both trials were carried out in Australia and the findings may not generalise to cultures where the stigma of disclosure is much higher. For example, it has been found that willingness to disclose is lower in Japan than in Australia (Griffiths *et al*., 2006). It is also possible that the findings could be different where the data were collected orally by an interviewer (as in many prevalence studies) compared to the online self-completion that was used in the current studies. In an interview situation with a stranger, stigmatising attitudes towards disclosure may play a greater role and participants may be more reluctant to report symptoms. Finally, the method used here to test the masking hypothesis assumes that the attitude changes that occur with MHFA training are similar in nature to those that might occur with historical change in the population.

## Conclusion

These findings do not support the hypothesis that increases in willingness to disclose mental health problems affect symptom scores. It is therefore unlikely that masking could be responsible for the lack of improvement in the mental health of the population following increased uptake of treatment.

## Data

Requests for access to the data should be made to the first author.

## Appendix

### Vignettes used in the adolescent MHFA trial

John is a 16-year-old who has been unusually sad and miserable for the last few weeks. He is tired all the time and has trouble sleeping at night. John doesn't feel like eating and has lost weight. He can't keep his mind on his studies and his marks have dropped. He puts off making any decisions and even day-to-day tasks seem too much for him. His parents and friends are very concerned about him. John says he will never be happy again and believes his family would be better off without him. John says he feels so desperate, he has been thinking of ways to end his life.

Jeanie is a 16-year-old living at home with her parents. Jeanie started at your school last year and you are the only friend she has made so far. She seems very shy and when you ask her why she doesn't make more of an effort, she says she would really like to make more friends but is scared that she'll do or say something embarrassing when she's around others. Although Jeanie's schoolwork is OK she rarely says a word in class and becomes incredibly nervous, trembles, blushes and seems like she might vomit if she has to answer a question or speak in front of the class. At her house you have seen that Jeanie is quite talkative with her family, but becomes quiet if anyone she doesn't know well comes over. She has stopped answering the phone and doesn't come to parties anymore. Jeanie says she knows her fears are unreasonable but she can't seem to control them and this really upsets her.

### Vignettes used in the adult MHFA trial

John is 30-years-old. He has been feeling unusually sad and miserable for the last few weeks. Even though he is tired all the time, he has trouble sleeping nearly every night. John doesn't feel like eating and has lost weight. He can't keep his mind on his work and puts off making decisions. Even day-to-day tasks seem too much for him. This has come to the attention of John's supervisor who is concerned about his lowered productivity. John feels he will never be happy again and believes his family would be better off without him. John has been so desperate, he has been thinking of ways to end his life.

Paula is a 30-year old office worker. Recently, her sleep has been disturbed and she has been having vivid nightmares. She has been increasingly irritable and can't understand why. She has also been jumpy, on edge and tending to avoid going out, even to see friends. Previously, she had been highly sociable. These things started happening around 2 months ago when she was assaulted and robbed by a man armed with a knife. This occurred while she was walking to the train station on her way to work. Paula sees the man's face clearly in her nightmares, but she won't talk about what happened. She now refuses to use public transport and this is affecting her work attendance.
